# Insights Into the Complexity of Craniofacial Development From a Cellular Perspective

**DOI:** 10.3389/fcell.2020.620735

**Published:** 2020-12-18

**Authors:** Andrea P. Murillo-Rincón, Marketa Kaucka

**Affiliations:** Max Planck Research Group Craniofacial Biology, Max Planck Institute for Evolutionary Biology, Plön, Germany

**Keywords:** craniofacial development, neural crest, mesenchymal condensations, chondrocranium, facial shape, signaling centers, cellular behavior

## Abstract

The head represents the most complex part of the body and a distinctive feature of the vertebrate body plan. This intricate structure is assembled during embryonic development in the four-dimensional process of morphogenesis. The head integrates components of the central and peripheral nervous system, sensory organs, muscles, joints, glands, and other specialized tissues in the framework of a complexly shaped skull. The anterior part of the head is referred to as the face, and a broad spectrum of facial shapes across vertebrate species enables different feeding strategies, communication styles, and diverse specialized functions. The face formation starts early during embryonic development and is an enormously complex, multi-step process regulated on a genomic, molecular, and cellular level. In this review, we will discuss recent discoveries that revealed new aspects of facial morphogenesis from the time of the neural crest cell emergence till the formation of the chondrocranium, the primary design of the individual facial shape. We will focus on molecular mechanisms of cell fate specification, the role of individual and collective cell migration, the importance of dynamic and continuous cellular interactions, responses of cells and tissues to generated physical forces, and their morphogenetic outcomes. In the end, we will examine the spatiotemporal activity of signaling centers tightly regulating the release of signals inducing the formation of craniofacial skeletal elements. The existence of these centers and their regulation by enhancers represent one of the core morphogenetic mechanisms and might lay the foundations for intra- and inter-species facial variability.

## Introduction

The shape of the face possesses species-specific features that give each vertebrate group its unique facial appearance. In some species such as humans, the otherwise conserved shaping mechanism is flexible enough to allow for shape fine-tuning, resulting in an impressive facial variability (Liu et al., 2012; Adhikari et al., 2016; Cole et al., 2016; Xiong et al., 2019). The shape of the face strongly depends on the geometry of underlying skeletal elements, adipose tissue, and muscles. While the postnatal facial skeleton in the majority of vertebrates is formed by bony elements, the embryonic face is built up entirely from cartilage and is represented by a complex structure called the chondrocranium. Most of the chondrocranium is substituted by the bone postnatally (McBratney-Owen et al., 2008; Kawasaki and Richtsmeier, 2017) and the functional and evolutionary meaning of chondrocranium is not fully understood. However, changes in chondrocranium morphology or morphometry remain even after its replacement by the bone (Kaucka et al., 2017), therefore, the formation of chondrocranium can be considered a key process in the acquisition of facial shape.

The chondrocranium formation is an intricate, multi-step process utilizing cells derived from all three germ layers and driven by, for instance, complex cell and tissue interactions, specific cellular behavior, or by a multitude of spatiotemporally active morphogens. In this review, we will summarize the advancements in our understanding of its developmental complexity. In the first part, we will focus on mechanisms driving the specification, delamination, and migration of cranial neural crest cells to their respective destinations, and their interactions with surrounding cells that result in sensory placode formation. Next, we will pinpoint to mutual interactions between different cell and tissue types, the cellular response to changing physical forces, and specific clonal behavior such as oriented cell division. In the end, we will expose the importance of the spatiotemporally defined activity of signaling centers driving the early craniofacial morphogenesis and discuss the possible genetic basis of facial variability.

## Specification and Cell Fate Acquisition of the Neural Crest Cell

**FIGURE 1:**
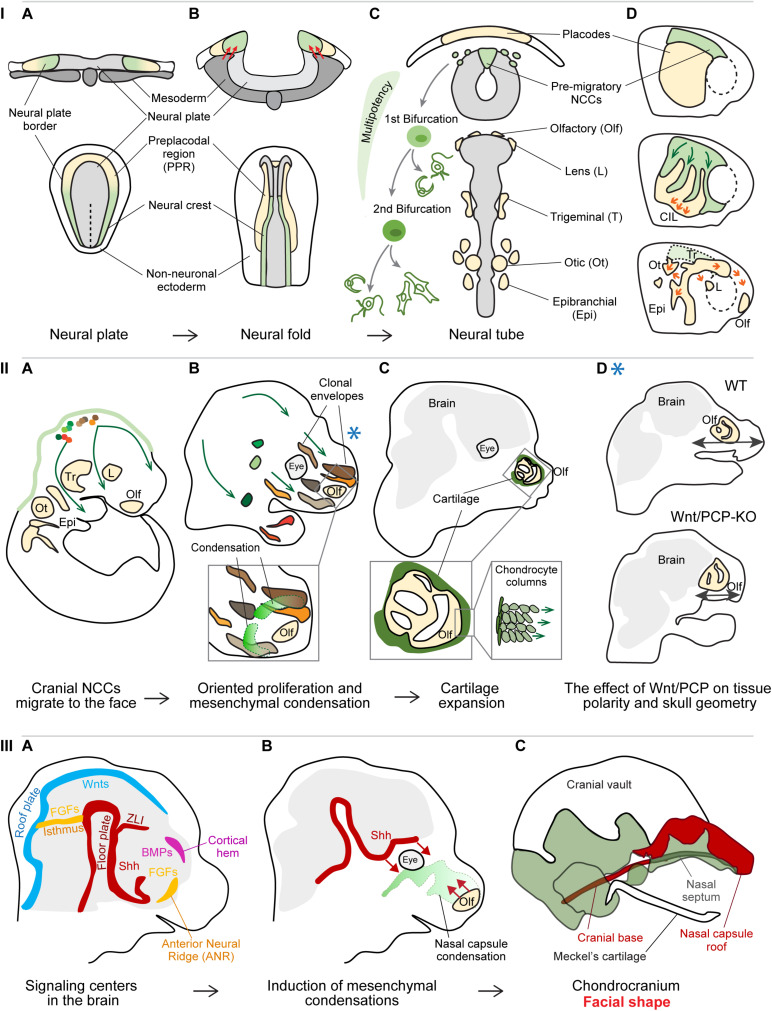
**Panel I:** Specification and cell fate acquisition of the neural crest cells (NCCs). **(A)** NCCs and placode progenitor cells are localized adjacent to each other at the neural plate border. **(B)** When neural folds are brought together, the stiffened mesoderm underlying the cranial NCCs (red arrows) triggers NCC EMT and initiate NCC migration. **(C)** The migrating NCCs undergo a gradual process of cell fate acquisition, during which the NCCs pass through two main bifurcation points. The first separation leads to the split of sensory and autonomic/mesenchymal fates, and the second separation leads to the emergence of autonomic and mesenchymal fates. Placode progenitors are split from the common PPR into individual placodes to give rise to different sensory organs or cranial ganglia. **(D)** NCCs migration streams and placode separation are a result of the interaction between NCCs and placode cells (middle): NCCs are attracted by placode cells (green arrows), after their direct contact placode cells are repolarized and move away from the NCCs (orange arrows), in a process called contact inhibition locomotion (CIL). Due to their active directed cell migration (orange arrows, below), the individual placode territories are established and NCCs had along their stereotypical migratory pathways. **(A–C)** Upper row transversal view, lower row: dorsal view; **(D)** lateral view. **Panel II:** Cellular behavior during early craniofacial morphogenesis. **(A)** The cranial NCCs migrated into their respective destinations and proliferate generating facial ectomesenchyme. **(B)** The progeny of each cranial NCC occupies a 3D area referred to as the clonal envelope. NCCs undergo cell divisions in an oriented manner, the shapes of clonal envelopes reflect the anisotropic growth of the head. The mesenchymal condensations originate from the local ectomesenchyme and represent the blueprint for chondrocranium and future facial shape. Extensive cell proliferation in quite distant locations results in collective cell movement translocating the whole micro-domains. **(C)** The mesenchymal condensations start differentiating into cartilage, the chondrocytes undergo a series of polarized cell divisions resulting in surface expansion of the chondrocranium. **(D)** Polarized cell division of NCC-derived ectomesenchyme is controlled by Wnt/Planar cell polarity (PCP) pathway and its disruption leads to shorter and wider facial proportions. The Blue asterisk shows the relevant time point of the Wnt/PCP signaling effect – during the proliferation of ectomesenchyme **(panel IIB)**. **Panel III:** Signaling centers establish facial geometry. **(A)** Signaling centers located in the developing brain instruct the formation of mesenchymal condensations from the ectomesenchyme. **(B)** Sonic hedgehog (Shh) from the floor plate induces the condensation leading to the formation of nasal septum as well some elements of the cranial base, while morphogenetic signals from the olfactory epithelium induce the formation of nasal capsule roof. **(C)** Removal of morphogenetic signals from distinct locations results in the absence or malformation of the respective cartilaginous structures.

A milestone in the process of head formation is the emergence and migration of the neural crest cells (NCCs). The NCCs are transient multipotent progenitors arising from the borders of the closing neural tube (Figures 1IA,B; Basch et al., 2006; Pla and Monsoro-Burq, 2018; Prasad et al., 2020). Depending on the location of their origin along the anterioposterior axis of the neural tube and their post-migratory target destination, the cells are classified as cranial (or cephalic), cardiac, vagal (including enteric), trunk, and sacral NCCs (Rothstein et al., 2018). After the NCCs specification, the cells undergo epithelial-to-mesenchymal transition (EMT), delaminate from the neural tube, and follow conserved stereotypical dorsolateral migratory streams (Figures 1IC,D; Minoux and Rijli, 2010; Theveneau and Mayor, 2012; Rothstein et al., 2018).

Interestingly, cranial and trunk NCCs utilize distinct migration strategies (Richardson et al., 2016). Trunk NCCs migrate as chains of single cells following the leader cells in the front of the streams. The leader cells manifest characteristic morphological properties that are observed already before the onset of migration and if ablated, the follower cells are unable to proceed ventrally pointing at the irreplaceable fixed role of the leader cell. Cranial NCCs move directionally as a whole and all the cells show similar migratory properties. All the cranial NCCs can serve as transient leaders and their ablation does not affect the migration of the following cells. Both cranial and trunk NCCs maintain continuous and dynamic cell-cell contact to keep migration and directionality (Richardson et al., 2016; Li et al., 2019). Analysis of the hierarchical clustering of NCCs in cyclostomes shed new light on the evolutionary origin of the cranial crest. The cranial subpopulation of lamprey NCCs showed transcriptional similarity to the gene signature of trunk NCCs in amniotes. These observations suggest that the NCCs underwent gradual acquisition of regulatory complexity during the evolution of vertebrates, leading to the existence of distinct NCC populations such as cranial and trunk, and likely affecting the vertebrate body plan (Martik et al., 2019).

The NCCs give rise to a broad spectrum of cell types such as neurons and glial cells of the peripheral nervous system, smooth muscles, endocrine cells, melanocytes, chondrocytes, odontoblasts, connective tissue, and bone (Bronner and LeDouarin, 2012; Baggiolini et al., 2015; Kaucka et al., 2016). In mouse, the analysis of the progenies (or clones) derived from genetically labeled single NCCs showed that each clone occupies a specific area in the forming head and has the potential to acquire diverse cell fates, therefore contributing to the formation of multiple structures in that particular location (Baggiolini et al., 2015; Kaucka et al., 2016). The impressive cell fate repertoire of the NCCs raised the question of how do NCCs generate and control their extraordinary multipotency and what are the molecular drivers of cell fate specification. Analysis of the pluripotency marker expression in cranial NCCs identified a neural crest stem cell niche located in the central-most portion of the dorsal neural tube (Lignell et al., 2017). Other NCC subpopulations positioned dorsolaterally to the stem cell niche expressed more canonical NCC markers or showed a typical migratory gene expression signature (Lignell et al., 2017). With increasing distance of NCCs from the central part of the neural tube, the effect of Wnt signaling coming from the neural tube decreases and results in the miRNA-mediated silencing of typical neural crest stem cell markers, leading to the loss of NCCs multipotency (Bhattacharya et al., 2018).

Epigenetic regulation is one of the further mechanisms controlling the cranial NCC plasticity, especially concerning their axial regional identity. The cranial NCCs maintain broad facial patterning competence and can contribute to different structures in the forming head. Cranial NCCs show similar chromatin accessibility patterns even though their transcriptional profile differs. The chromatin pattern is maintained until post-migratory stages when the cells commit to a specific fate in response to local cues (Minoux et al., 2017). In Xenopus, the activity of histone deacetylases (HDACs), enzymes regulating chromatin accessibility, was shown to be crucial in blastula cells for patterning the ectoderm and the establishment of NCCs, and in the NCCs to maintain their pluripotency (Rao and LaBonne, 2018).

The combination of the single-cell transcriptomics and bioinformatical analysis of branching trajectories of murine NCCs, revealed new aspects of NCC multipotency, EMT, and fate specification (La Manno et al., 2018; Soldatov et al., 2019). Recent findings disproved previous beliefs that these are abrupt events driven by the activation of a specific gene regulatory network or dependent on the expression of a master regulator gene. For instance, pre-migratory NCCs express both neural plate border-specific genes and several neural tube markers (Lignell et al., 2017; Soldatov et al., 2019; Williams et al., 2019). The expression of NCC-specific markers gradually outweighs the expression of neural tube-specific genes and the NCCs initiate delamination from the neural plate. The further existence of migrating NCCs is also defined by an extensive sequence of transcriptional events (Soldatov et al., 2019). Specifically, cell fate commitment is a result of co-activation of gene expression programs that would normally lead to mutually exclusive cell fates. Their competition is resolved after a bifurcation point, which is likely reached due to the effect of extrinsic signal such as WNT, BMP, Notch, or another signaling (Figure 1IC). Before the cell passes the bifurcation point, it can be perceived as bipotent progenitor. Interestingly, the first bifurcation point leads to division of progenitors into the sensory and autonomic neuronal/mesenchymal lineage, followed by the subsequent separation of autonomic neuronal progenitors from mesenchymal lineage (Figure 1IC; Soldatov et al., 2019), which is contrary to previous belief that each type of progenitor is directly derived from one common precursor.

## Cell and Tissue Dynamics in Early Craniofacial Morphogenesis

Migration of single cells and large-scale collective cell movements represent important aspects of early facial morphogenesis. Such movements enable both mechanical and molecular interactions between cells and tissues, and their environment. One of the examples of such coordinated movement are the reciprocal interactions between the cranial NCCs and cranial placode cells. Cranial placode precursors arise at the anterior part of the neural plate border, and form a common pre-placodal region (PPR) characterized by the expression of the transcription factor Six1 (Figure 1IA). The PPR is positioned in close proximity and laterally to the emerging neural crest cells (Sato et al., 2010; Saint-Jeannet and Moody, 2014; Riddiford and Schlosser, 2016; Roellig et al., 2017). The cranial placodes together with the cranial NCCs form specialized sensory organs in the vertebrate head (Figure 1IC; Bouchard et al., 2010; Ladher et al., 2010; Huang et al., 2011; Cvekl and Ashery-Padan, 2014). The separation of common PPR into individual placodes is closely related to NCCs migration (Figure 1ID). Delaminating NCCs are attracted by the chemokine Sdf1 (CXCL12) produced by the placode cells (Shellard and Mayor, 2019). Once the NCCs get in direct contact with placodal cells, a transient junction complex is formed and Wnt/PCP components together with N-cadherin signaling trigger contact inhibition locomotion (CIL) (Theveneau et al., 2013). The cell protrusions and focal adhesions of placodal cells at the region of contact collapse, which leads to their repolarization and the directional migration away from the NCCs. Such “chase and run” behavior of NCCs following the Sdf1-producing placodal cells results in both the physical separation of future placodal regions and the establishment of the NCCs the migration streams (Figure 1ID; Szabó et al., 2019).

The cranial NCCs then follow stereotypical migratory streams and proliferate on their way to finally settle in various compartments of the head and produce ectomesenchyme (Figure 1IIA; Minoux and Rijli, 2010; Kaucka et al., 2016; Rothstein et al., 2018). While active and directional cell migration rather than cell proliferation drives the formation of the olfactory, lens, and otic placodes (Steventon et al., 2016; Breau et al., 2017), the ectomesenchyme expands by series of cell divisions and only very limited individual cell migration (Kaucka et al., 2016). A single NCC clone ultimately occupies a certain 3D space, referred to as a clonal envelop (Figure 1IIB). The shape and size of the clonal envelopes mirror the local directional cell behavior (the extent and the polarity of cell division), the level of mixing with other NCC clones, and reflect the anisotropic growth of different regions in the head. Each location is populated by several NCC-derived clones that extensively mix and jointly contribute to the formation of different structures in that area (Figure 1IIB). This might represent a compensation mechanism in the case that one or a few NCCs would be eliminated by, for instance, negative somatic mutations (Kaucka et al., 2016).

Functional experiments targeting different components of the Wnt/PCP signaling highlighted the importance of the ectomesenchyme polarity for the proper facial outgrowth in several developmental time-points. Overall, the rate of cell divisions in Wnt/PCP mutants does not appear to be affected, however, the impaired direction of daughter cell allocation during cell division results in a variety of pathologies, such as shorter and wider facial proportions, abnormal mesenchymal patterning during early palatogenesis or misoriented and misshaped chondrocytes affecting the facial morphometry (Figures 1IIC,D; Le Pabic et al., 2014; Brock et al., 2016; Kaucka et al., 2016). Oriented deposition of daughter cells also represents a core elongation mechanism driving the longitudinal growth of flat facial cartilage composing the mammalian chondrocranium (Figure 1IIC; Kaucka et al., 2017). Normal facial morphogenesis furthermore relies on coordinated collective cell movements, where the shift of large cell masses resembles the behavior of viscous fluids and allow large-scale cell rearrangements (David et al., 2014; Méhes and Vicsek, 2014; Kaucka et al., 2016; Tao et al., 2019). These crowd movements are driven by extensive cell proliferation in relatively remote regions positioned laterally or posteriorly to the respective area and allow the preservation of cellular arrangements of the pushed microdomains (Kaucka et al., 2016).

Furthermore, physical forces generated by morphogenetic events contribute to various aspects of head formation. Cranial mesoderm stiffening was shown to be necessary and even sufficient to induce NCCs EMT and migration in *Xenopus* (Figure 1IB; Barriga et al., 2018). The stiffness of the cranial mesoderm, underlying the cranial NCCs at the time of neurulation, is generated by the increase in cell density in the process of convergent extension. The NCCs use their integrin-vinculin-talin mechanosensory complex to perceive the mechanical changes in the environment, and respond by switching the expression of E-cadherin to N-cadherin, gaining motility and responsiveness to chemotactic cues (Scarpa et al., 2015; Barriga et al., 2018). Mechanical forces also drive tooth morphogenesis (Mammoto et al., 2011; Panousopoulou and Green, 2016). The invagination of epithelial thickening into the mesenchyme is driven by the physical contraction of superficial layers of cells (i.e., suprabasal cells) (Panousopoulou and Green, 2016). The contraction is generated by the suprabasal cells intercalating and extending their cell shape apically and centripetally while remaining attached to the basal lamina. This process appears to be evolutionary conserved and may represent a fundamental morphogenetic mechanism driving the formation of multiple ectodermal organs such as a tooth, hair follicle, mammary gland, and sweat glands (Panousopoulou and Green, 2016). Subsequently, mesenchymal cells are attracted to underneath the early dental epithelium, which results in their physical compaction (Mammoto et al., 2011). Such mesenchymal cell condensation in turn triggers the induction of odontogenic expression programs and cell fate specification.

## Establishing the Facial Geometry: The Mesenchymal Condensations

The first solid geometrical layout of the future facial shape is represented by the chondrocranium, the cartilaginous template of the future skull. The chondrocranium is induced when the ectomesenchyme formed the frontonasal outgrowth. A key step preceding the formation of cartilage is the induction of mesenchymal condensations (Figure 1IIB). These are defined areas of mesenchyme that are compacted due to local rapid cell division (Kaucka et al., 2017, 2018; Giffin et al., 2019), and differentiate into cartilage shortly after their emergence (Figure 1IIC). The spatially defined sources of morphogenetic signals, often represented by secreted ligands with long-range action potential (e.g., WNTs, FGFs, BMPs, and Hedgehogs), are referred to as signaling centers or organizers (Figure 1IIIA; Martinez Arias and Steventon, 2018; La Manno et al., 2020). The known signaling centers involved in craniofacial morphogenesis are located in the emerging nervous system, in the foregut endoderm and in spatially defined areas of the facial ectoderm (Marcucio et al., 2005; Wada et al., 2005; Brito et al., 2006; Eberhart et al., 2006; Foppiano et al., 2007; Szabo-Rogers et al., 2008, 2009; Gitton et al., 2011; Reid et al., 2011; Bhullar et al., 2015; Kaucka et al., 2018; Crane-Smith et al., 2019). Tissue- and/or time-specific ablation of morphogens or their sources, such as Sonic Hedgehog (Shh) or the olfactory placodes, showed that the condensations, and in turn the cartilage, are induced by different signaling centers at distinct time points as independent units, pointing at the composite character of the chondrocranium and the future skull (Figures 1 – IIIB, C; McBratney-Owen et al., 2008; Kaucka et al., 2018). Based on the association of the positions of both the signaling centers and skeletal structures induction points (Fabbri et al., 2017), it has been proposed that the species-specific localization of signaling centers located in the brain may represent a reliable criterion to assess skull bone homology (Teng et al., 2019). The Frontonasal Ectodermal Zone (FEZ) of birds and mammals represents a signaling center in the frontal facial ectoderm secreting SHH and FGF8, coordinating ectomesenchyme behavior and affecting the size, position, shape, and orientation of mesenchymal condensations (Abzhanov et al., 2007; Foppiano et al., 2007; Hu and Marcucio, 2009; Hu et al., 2015a,b).

Both the position of the signaling centers in the developing head and the expression levels of the inductive signals can be modified by several mechanisms. One of them is represented by the cis-regulatory elements (i.e., enhancers) that alter the levels of gene expression. Deletion of enhancers or changes in their sequence can result in a wide range of facial phenotypes (Attanasio et al., 2013; Kaucka et al., 2018). The sequence variation found in regulatory elements of genes associated with facial shape variability and identified epigenetic modifications controlling the enhancer activity in humans (Claes et al., 2018), show that enhancers represent an exceptionally flexible mechanism allowing facial shape fine-tuning (Kaucka et al., 2018) and may account for one of the mechanisms behind intra- and/or inter-species facial differences. Understanding the basis and regulation of mesenchymal condensation induction is further important for the comprehension and management of human craniofacial syndromes. More than 30% of all congenital abnormalities are represented by craniofacial malformation, ranging from subtle changes of facial features or symmetry to severe conditions affecting feeding, breathing, or other aspects of survival (Xavier et al., 2016; Abramyan, 2019). Interestingly, human craniofacial syndromes are often associated with disorders affecting the central nervous system or the sensory organs (Marcucio et al., 2011; Twigg and Wilkie, 2015), which further underlines the conserved link between the development of the nervous system and the formation of chondrocranium.

## Discussion

The development and availability of new techniques in the last decade have significantly advanced our understanding of the complexity of craniofacial development and opened new perspectives for future research. Single-cell omics analysis can be applied to uncover the gene regulatory networks driving the transitions from mesenchyme to cartilage or to reveal the enhancer-driven control of gene expression levels in different cellular populations. This approach, in combination with genetic tracing and live imaging, can further dissect the interactions between various cell or tissue types in detail and resolve, among others, the still enigmatic processes of placode specification and individualization. The precise spatiotemporal mapping of the signaling centers in the developing embryos of various model organisms and exploring their genomic regulation will advance our understanding of both the intra- and inter-species facial variability and the genetic basis of facial features heritability. Additionally, such investigation will allow us to better comprehend the nature of rare craniofacial pathologies. Further research of the conserved developmental link between the formation of the nervous system and the skull may identify new therapeutic targets to reduce the impact of severe congenital syndromes manifested with craniofacial deformities or offer new strategies to enhance cartilage regeneration in adulthood.

## Author Contributions

AM-R and MK wrote the manuscript and designed the figure. Both authors contributed to the article and approved the submitted version.

## Conflict of Interest

The authors declare that the research was conducted in the absence of any commercial or financial relationships that could be construed as a potential conflict of interest.
